# Supplementary material for: Causal Relationship between Aging and Anorexia Nervosa: A White-Matter-Microstructure-Mediated Mendelian Randomization Analysis
Source: Biomedicines. 2024 Aug 16;12(8):1874. doi: 10.3390/biomedicines12081874 (PMC11351342; doi:10.3390/biomedicines12081874)
Supplement: Supplementary file 1 [file biomedicines-12-01874-s001.zip › Supplementary Figure/Figure S4/Singe SNP plot of MR analysis for TL to ubm-b-1828.pdf]

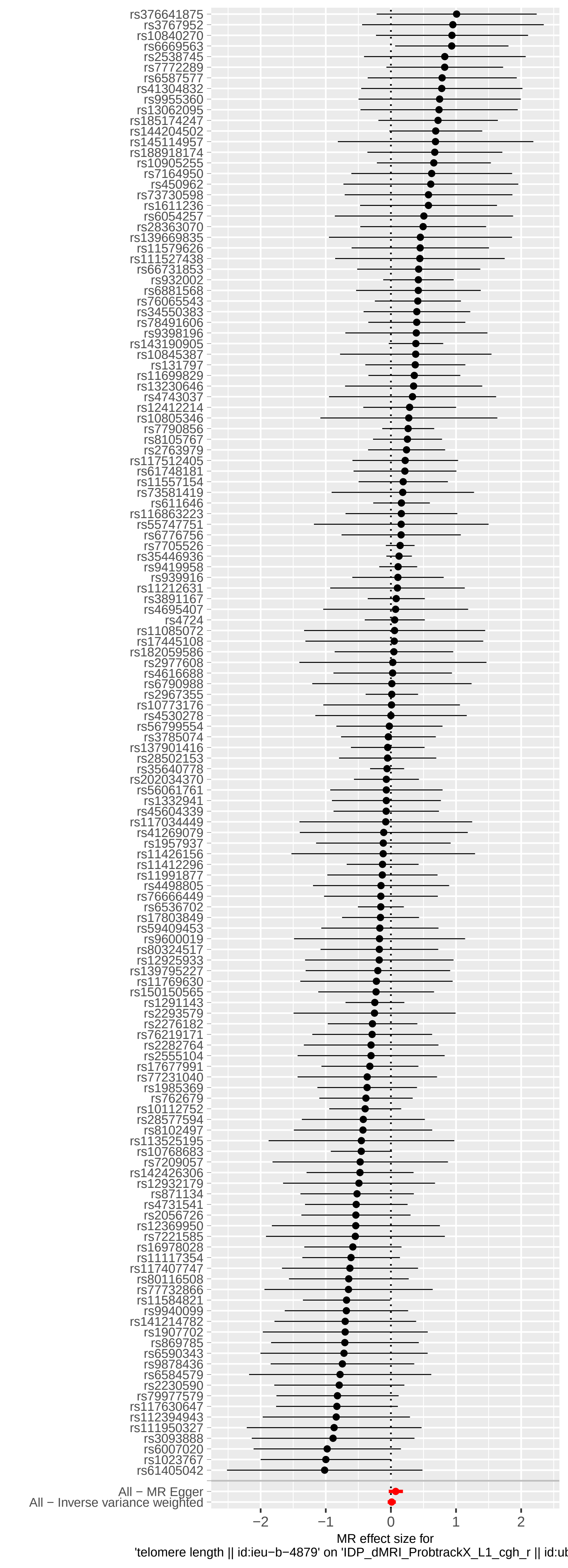
rs376641875
rs3767952
rs10840270
rs6669563
rs2538745
rs7772289
rs6587577
rs41304832
rs9955360
rs13062095
rs185174247
rs144204502
rs145114957
rs188918174
rs10905255
rs7164950
rs450962
rs73730598
rs1611236
rs6054257
rs28363070
rs139669835
rs11579626
rs111527438
rs66731853
rs932002
rs6881568
rs76065543
rs34550383
rs78491606
rs9398196
rs143190905
rs10845387
rs131797
rs11699829
rs13230646
rs4743037
rs12412214
rs10805346
rs7790856
rs8105767
rs2763979
rs117512405
rs61748181
rs11557154
rs73581419
rs611646
rs116863223
rs55747751
rs6776756
rs7705526
rs35446936
rs9419958
rs939916
rs11212631
rs3891167
rs4695407
rs4724
rs11085072
rs17445108
rs182059586
rs2977608
rs4616688
rs6790988
rs2967355
rs10773176
rs4530278
rs56799554
rs3785074
rs137901416
rs28502153
rs35640778
rs202034370
rs56061761
rs1332941
rs45604339
rs117034449
rs41269079
rs1957937
rs11426156
rs11412296
rs11991877
rs4498805
rs76666449
rs6536702
rs17803849
rs59409453
rs9600019
rs80324517
rs12925933
rs139795227
rs11769630
rs150150565
rs1291143
rs2293579
rs2276182
rs76219171
rs2282764
rs2555104
rs17677991
rs77231040
rs1985369
rs762679
rs10112752
rs28577594
rs8102497
rs113525195
rs10768683
rs7209057
rs142426306
rs12932179
rs871134
rs4731541
rs2056726
rs12369950
rs7221585
rs16978028
rs11117354
rs117407747
rs80116508
rs77732866
rs11584821
rs9940099
rs141214782
rs1907702
rs869785
rs6590343
rs9878436
rs6584579
rs2230590
rs79977579
rs117630647
rs112394943
rs111950327
rs3093888
rs6007020
rs1023767
rs61405042
All − MR Egger
All − Inverse variance weighted
−2
−1
0
1
2
MR effect size for
'telomere length || id:ieu−b−4879' on 'IDP_dMRI_ProbtrackX_L1_cgh_r || id:ub
